# Allonymous science: the politics of placing and shifting credit in public-private nutrition research

**DOI:** 10.1186/s40504-020-00099-y

**Published:** 2020-06-22

**Authors:** Bart Penders, Peter Lutz, David M. Shaw, David M. R. Townend

**Affiliations:** 1 Department of Health, Ethics & Society, Care and Public Health Research Institute (CAPHRI), Maastricht University, PO Box 616, Maastricht, NL-6200 MD the Netherlands; 2 School of Information Technology, Halmstad University, Halmstad, Sweden; 3 Institute for Biomedical Ethics, University of Basel, Basel, Switzerland

**Keywords:** Authorship guidelines, Research organisation, Governance, Renaissance, Allonymous science, Ghost authorship, Ghost collaboratorship

## Abstract

Ideally, guidelines reflect an accepted position with respect to matters of concern, ranging from clinical practices to researcher behaviour. Upon close reading, authorship guidelines reserve authorship attribution to individuals fully or almost fully embedded in particular studies, including design or execution as well as significant involvement in the writing process. These requirements prescribe an organisation of scientific work in which this embedding is specifically enabled. Drawing from interviews with nutrition scientists at universities and in the food industry, we demonstrate that the organisation of research labour can deviate significantly from such prescriptions. The organisation of labour, regardless of its content, then, has consequences for who qualifies as an author. The fact that fewer food industry employees qualify is actively used by the food industry to manage the credibility and ownership of their knowledge claims as allonymous science: the attribution of science assisted by authorship guidelines blind to all but one organisational frame.

## Introduction

One of the ways in which scientific communities attempt to foster responsibility amongst its members is through guidelines. Guidelines reflect an accepted position on matters of concern, ranging from clinical practices to researcher behaviour. Often, but certainly not always, guidelines are drafted by independent experts or groups of experts. In the context of authorship guidelines, those set forth by the International Committee of Medical Journal Editors (ICJME 2019) are most commonly referred to. Although these guidelines originate from a collective of (bio) medical experts, they have travelled well beyond the boundaries of medicine. Guidelines in general and the ICMJE authorship guidelines in particular draw legitimacy, on the one hand, from their relative independence of power structures governing practices including the governance of biomedical research. On the other hand, their legitimacy rests upon the recognised expertise of those who drafted them and, consequently, their accepted membership of the scientific communities the guidelines serve.

Legitimacy is a requirement for guidelines to have any effect on practices. The association of legitimacy and community membership of the guidelines’ authors is supposed to ensure that the explicit and tacit norms and values that govern authorship practices be embedded within the guidelines. Ideally, the communities targeted by guidelines view them as feasible, sensible, and appropriate. However, in the case of authorship guidelines, there actually is little evidence that they have significantly helped alter researcher behaviour (Kornhaber et al. 2015). In their review of authorship issues, Kornhaber et al. note that we do not know whether the lack of influence of the ICJME authorship guidelines is a result of their poor articulation or implementation, whether they are poorly enforced or enforceable, or whether the guidelines are clear but actively ignored by authors. The consequence is that the legitimacy and authority of authorship guidelines continue to be debated in research practices.

In a large quantitative analysis, Fong and Wilhite (2017) conclude that honorary authorships remain common across the sciences. Similarly, a smaller qualitative study revealed that researchers in nutrition science attach relatively little value to formal authorship criteria or guidelines, and adhere to local norms and conventions instead (Penders 2017a). Louis et al. (2008) show how various local, contextual factors are of great importance in authorship decisions, and that these decisions are often reached against a background in which authorship is primarily a commodity. Based on a thought experiment, Shaw (2011) has argued that the ICMJE criteria are illogical and unethical; MacFarlane et al. (2017) have repeated a similar point in the form of a scenario. Shaw and MacFarlane show that the guidelines allow significant contributions either to go unacknowledged, or do not help resolving disagreements should they occur. The requirement of ‘substantial’ contributions, as ICMJE criteria require, is not made explicit, and Ivaniš et al. (2008) have shown that whether this is understood as a binary or ordinal variable produces significant differences in the perception of legitimate authorship attribution. Such disagreements often lead to struggles, which Kovacs (2017) has described as a form of symbolic violence. Despite the existence of authorship guidelines, determining and agreeing upon authorship distribution continues to be a process that is unstable, vague, and subject to pre-existing power distributions. Consequently, it suffers from a potential disconnect between credit and responsibility (Biagioli 1998, 2000; Marušić et al. 2011; Street et al. 2010; Penders 2017b; Helgesson and Eriksson 2018; Schuyt 2019).

The organisation of scientific work has occupied science studies scholars for decades (Hackett et al. 2017). In particular, seminal laboratory studies in biology and physics (Knorr-Cetina 1981, 1995; Latour and Woolgar 1979; Lynch 1985; Traweek 1988) have foregrounded the conceptualisation of science as work, and have made the organisation of that work a relevant object of study in the context of the knowledge production enterprise. The organisation of scientific work is a moving target. It changes and evolves as science’s objects of study change. Linked with this are the changing requirements of governments and funders, which pressure scientific policies and practices. The guidelines and criteria prescribing responsible science evolve in a similar fashion. The recent focus on open science and open data is an example of how changing technical capabilities (data storage, curation and handling), social and political pressures (on efficient use of public funding by promoting reuse and replicability) and prescriptions in science (through funding requirements and data guidelines) co-evolve (Leonelli 2013; Mirowski 2018; Nosek et al. 2015). Authorship guidelines are no exception. These also exist in a context of evolving and plural practices in the organisation of scientific work as a whole.

In this paper, we first ask whether dominant authorship guidelines, and the ICMJE guidelines in particular, embody a conceptualisation of scientific practice that aligns with the governance of research in non-academic settings. Second, we consider the consequences of such a misalignment. In other words, who is the imagined scientist or what is the imagined organisation of scientific work written into the ICMJE authorship guidelines, and how do they compare with examples of actual scientists and actual scientific work? In addition to guidelines reasonably conceptualising actual practices, guideline compliance is heavily influenced by forces shaping publication practices in general. Quite prominently, these include the influence of evaluation mechanisms, such as publication-based performance metrics in academic institutions that amplify the need to pursue authorships, pursuing or avoiding collaborative publishing, and engaging in questionable behaviour to accomplish these (Hangel and Schmidt-Pfister 2017; Fochler et al. 2016). However, these external influences exist in addition to the potential misalignment between an imagined and actual scientist, and the imagined and actual organisation of scientific work. In this text, we do not set out to offer clear prescriptive measures or concretely revise authorship guidelines. Rather, we aim to expose the implicit problem of what we will call “allonymous science” in order to render the problem more explicit and allow a debate that includes those who operate on the boundary between the public and private spheres. In what follows, we first engage in a close reading of the most recent ICMJE guidelines to identify a few of the key characteristics of the imagined scientist and the imagined scientific work she or he embodies. Subsequently, we demonstrate empirically that the actual scientist and especially the actual organisation of scientific work differ from their imagined counterparts, especially (but not exclusively) when we look beyond the confines of the university. We conclude that this mismatch allows for the rendering invisible (or less visible) of corporate contributions to published works.

## Approach

In order to map how the imagined organisation of scientific work can be superimposed upon actual practices, we report on an empirical study based on two series of interviews held with nutrition scientists. These studies - targeting research integrity, authorship, value and credit in nutrition science - took place in 2016 and 2019. The first study consisted of 16 interviews mapping authorship issues. In addition, it explored the organisation of scientific work in the sense that legitimating authorship and authorship decisions always took place with explicit reference to work performed, responsibilities for that work and relationships that help distribute work. Interviewed researchers were all engaged in publication practices but ranged from early career researchers (ERCs) to senior researchers. The respondents were distributed across universities, contract research organisations and food companies (ranging from small consultancy companies to large multinationals). All respondents were members of the Dutch Academy of Nutrition Science (NAV).^1^

While the first interview series (2016) and its analysis allowed us to distinguish between a large number of salient differences in the organisation of work across institutional types, we lacked a dataset that allowed actor comparisons. To remedy this, we conducted a second interview series (2019), drawing again from the membership pool of the NAV. This time we focused on the identification of respondents who had recently switched from academic to commercial careers (or back), or whose careers were characterised by multiple switches. Table 1 lists key attributes of all respondents.

**Table 1:** Respondents

Interview Code	Organisation Type (current employment)	Gender	Seniority	Year
**IM1**	Industry	F	Junior	2016
**IW1**	Academia	F	Junior	2016
**IW2**	Academia	F	Junior	2016
**IT1**	CRO	F	Senior	2016
**IV1**	Industry	M	Senior	2016
**IU1**	Industry	M	Senior	2016
**IU2**	Industry	M	Mid-career	2016
**IW3**	Industry	F	Mid-career	2016
**ID1**	CRO	F	Mid-career	2016
**IT2**	CRO	F	Junior	2016
**IW4**	Academia	F	Senior	2016
**IS1**	Academia	F	Mid-career	2016
**IS2**	Academia	F	Senior	2016
**IB1**	Industry	M	Senior	2016
**IB2**	Industry	M	Mid-career	2016
**IB3**	Academia	M	Junior	2016
**IM2**	Academia	M	Senior	2019
**IA1**	Industry	F	Mid-career	2019
**IZ1**	Industry	F	Junior	2019
**IW5**	Industry	F	Junior	2019
**IG1**	Industry	M	Mid-career	2019

The analysis was informed by a series of sensitising concepts drawn from previous work and existing literature on authorship and the organisation of scientific work (Bowen, 2006). Using these sensitising concepts helped tailor the interview topic list to the research questions and helped structure the analysis of the interview transcripts. These were (1) collaborative working relationships (Bezuidenhout 2017; Müller 2012; Penders et al. 2015; Vermeulen et al. 2013; Shrum et al. 2001, 2007), (2) task distribution, uncertainty and interdependence (Cummings and Kiesler 2005; Fochler and Sigl 2018; Walsh and Lee 2015; Whitley 2000), (3) negotiations of professional autonomy (Fowler 2006; Hagstrom 1964; Parker et al. 2010), (4) incentives, rewards and (e)valuations (Hammarfelt et al. 2016; Rushforth and de Rijcke 2015; Hangel and Schmidt-Pfister 2017; Müller and de Rijcke 2017) and (5) articulations of ‘good science’ in the context of authorship (Biagioli 1998; Kovacs 2017; Marušić et al. 2011; Shaw 2011; Street et al. 2010).

## Results

### The imagined organisation of scientific work

In our analysis, we find that authorship guidelines are written in ways that assume and idealise the scientist and nature of scientific work. We interpret this as a largely renaissance imaginary that depicts the scientist as a fully autonomous individual. He or she performs all or nearly all of tasks of knowledge production - from involvement with its design, analysis and documentation to continued commitment once the work is published. This means that while on the epistemic level a lot of specialisation takes place, this is far less the case on the level of daily tasks. This implies competition and construes collaboration as a free choice. We recognise this renaissance articulation of academia beyond authorship too. Academics are expected to be excellent researchers, excellent teachers, excellent supervisors, excellent managers, excellent leaders, excellent disseminators, excellent coaches, et cetera (Van den Brink and Benschop 2012). Even the so-called storybook images of scientists (Veldkamp et al. 2017) do not set standards as high and diverse as those expected by universities.

The ICMJE guidelines are the paradigmatic example of this renaissance requirement for researchers, especially in the context of nutrition research (and the life sciences in general) where they act as a gatekeeper for credit distribution.^2^ They set in stone (or at least in clay, given occasional revisions) the need to make a substantial contribution - not only at one point but at virtually all stages of a research project:

Substantial contributions to the conception or design of the work; or the acquisition, analysis, or interpretation of data for the work; AND

Drafting the work or revising it critically for important intellectual content; AND

Final approval of the version to be published; AND

Agreement to be accountable for all aspects of the work in ensuring that questions related to the accuracy or integrity of any part of the work are appropriately investigated and resolved. (ICJME 2019)

The first criterion here means that all researchers, or at least all those who seek to be listed as authors, must be involved in either conceptualising or designing the project, or acquiring and analysing or interpreting data. However, many researchers will not be in a position to be involved in design or acquisition, having been hired only after funding was obtained (long after the project was designed). Similarly, there is no guarantee that those involved in the design phase will still be around in the analysis phase, with precarious employment conditions restricting employment - more senior research designers might never step into the lab anymore given the weight of other (especially administrative or leadership) responsibilities. Despite this, this first criterion is easier to meet than the following one, at least formally, as most researchers on empirical projects will be involved in gathering or working on data, or both, even if only minimally.^3^

The second criterion directly addresses the issue of writing. It again has a crucial “or”- a researcher must be involved in drafting the paper itself, ***or*** revising the paper for important intellectual content. This latter phrase remains undefined, which is problematic. Often one author will write the first draft alone. This means that any other authors must all individually make an important intellectual contribution or be excluded from authorship, even if she or he conducted most of the data collection and analysis. Indeed, if the initial analysis was excellent and the first draft was good, there might not be a great need to revise it in a way that meets the “important” criterion. More significantly, it could be argued that one could write a first draft without contributing any important intellectual content if one had good data and analyses with which to work. Furthermore, researchers can disagree over what constitutes an important contribution, and this criterion is central to many practical and conceptual disagreements.

The third criterion might seem relatively benign. One might assume that it is a simple requirement to let all authors see the final version before it is submitted to a journal. In practice, however, this is unlikely to be the case. When the final version is shared with all researchers on a project, junior researchers are usually not in a position to refuse to approve or accept it because of the power dynamics in research teams. Furthermore, it is often at this ‘final’ stage that the authorship order is stated on the paper for the first time, often without prior discussion (Kovacs 2017; Penders 2017a). The key part of the ICMJE guidelines make no reference to author order explicitly. The final version of any paper will always include the author sequence, meaning that all authors have to endorse it even if they disagree with it. This is particularly problematic because it is at this stage that junior researchers are sometimes omitted or ‘moved down’ in the author order or guest authors suddenly appear (Kovacs 2017; Macfarlane et al. 2017).

Similar issues affect the fourth and last criterion. Researchers can certainly be willing to cooperate with any investigation into the integrity of a project in which they were involved, but they can hardly be held accountable for all aspects of the work if they were not involved in all aspects of the work. In addition, most researchers will be well aware of the potential consequences of calling into question the work of their colleagues.

There is also a supplemental requirement described below the main criteria: “In addition to being accountable for the parts of the work he or she has done, an author should be able to identify which co-authors are responsible for specific other parts of the work. In addition, authors should have confidence in the integrity of the contributions of their co-authors” (ICJME 2019). On a small project, it is reasonable to expect researchers to know exactly who did what, but on a large multi-centre project, this is an unrealistic requirement. In terms of integrity, it is not clear how researchers can be expected to have confidence in the contributions of researchers that they have never worked with or perhaps even met. Another supplemental requirement states that “The individuals who conduct the work are responsible for identifying who meets these criteria and ideally should do so when planning the work, making modifications as appropriate as the work progresses” (ICJME 2019). This is also problematic for multi-sited studies, where there is a high degree of trust involved that the research leaders at other institutions are only including those who meet the criteria. But it is also problematic in terms of power - junior researchers can hardly be expected to determine and dictate who should and should not be authors on a given paper if their professors disagree, even if they should certainly be involved in such a process.

This brief overview of the ICMJE authorship guidelines already gives some idea of the potential problems that await their real-world application. Regardless of whether ICMJE criteria are interpreted as binary or ordinal (Ivaniš et al. 2008), requiring involvement in all facets of knowledge production raises the question of whether researchers really are renaissance women and men.

### The organisation of scientific work

As previously demonstrated, nutrition scientists have diverse views on the role and value of authorship, or the pursuit of it, for the benefit of their work and careers. This diversity roughly aligns with their institutional affiliations, most notably through reward structures and the institutional goals reflected in them (Penders 2017a). These produce different ways of reflecting team contributions in team authorships. This and other previous work did not link the organisation of knowledge production labour (science as work) to authorship pursuits and authorship allocation practices.

Most respondents (18 out of 21) argue that contributing to the production of research publications is part of their job. The audience of those research publications can differ, ranging from potentially everyone (scientific publications in open access journals) to a select audience within the author’s organisation. How they see and value their contribution and what institutional characteristics help determine what that contribution can look like is, however, remarkably diverse.

#### Renaissance pursued

In academia, the organisation of work will be recognisable to most readers, even if there is remarkable diversity in how the distribution of work is organised and how concrete tasks are divided. Respondents took ownership of specific sets of tasks - in the case of junior researchers, sets of tasks were assigned to them. Consider this postdoc who reflects on the start of her PhD thesis work:

Well, eh, we had a PhD research project that actually written by four supervisors. I applied for it and was selected. So, eh, yeah, a lot of the thinking was already done. I mostly did the execution. I worked with epidemiological data and most of that was already collected, so I only had to… I just got the data and started analysing. When I was done, I started writing. But I also did [another] study, which I designed and performed myself. I was working with participants and yeah… that took two to three years. Interview IW2.

Researcher IW2 displays that, while in some situations actions like research design, data collection and data analysis may be separated (her first initiation into her thesis work), she was nonetheless expected to perform all those tasks herself at a later stage. While thesis work is often, but not always, considered “small” science, even in the context of “big” or well-funded science, researchers expect of themselves that they take upon them a diverse set of tasks:

For a big study, […] I am the first author, together with […]. This was a research that I coordinated myself fully, I wrote the protocol, I wrote the report, I did the work, I managed and did the whole thing. It made perfect sense that I would also write the article and I did. I did it with a lot of colleagues, because it is a complex piece, not completely my expertise, so it made sense to involve others too. Interview IT1.

Even in the context of teamwork, in which multiple expertises are required to complete a single project, researcher IT1 is involved directly with a variety of tasks and a variety of elements in the organisation of scientific labour: planning, designing, writing, executing, and managing. In the context of these larger projects others were involved as well, presumably for most if not all of those tasks. The upscaling of research resulted in more hands working on the project, but not in researcher IT1 withdrawing from certain tasks.

This, of course, does not mean that every researcher in every lab is always involved in every task. Earlier work on authorship in nutrition science already demonstrated that especially for “middle authorships”, very little involvement was expected from senior academics (Penders 2017a). Nonetheless, key authorship positions still required involvement in tasks represented on all levels in the ICJME authorship guidelines. This is also built into how individual scientists identify and seek out tasks for themselves, in the sense that prospective authorship stimulates them to adhere to the many tasks and types of work it requires, for instance by engaging with draft texts critically and providing detailed feedback.

The assumptions about how science is organised and what scientists do, written into the ICMJE authorship guidelines do not ideally fit the reality of precarious labour conditions, the informal organisation of work, or the pursuit of reputation as observed in universities. Nonetheless, these very same assumptions about what research is supposed to look like do exist there, and thereby shape actual research and publication practices.

#### Renaissance undressed

The organisation of work in the food industry differs from that in academia. Again, those differences are not uniform and not every company adheres to the same organisation models, types of workflow and more. The distribution of tasks across people in a company or in a single department within a company can be specified in detail – even though that does not mean that it is fully stable, as researcher IZ1 explains:

We are growing hard now, so people join us all the time. Every time that happens, our individual duties shift, just enough to squeeze in the new person. Sometimes it is a very specific job that is to be filled and sometimes support for the whole team. When it happens, we have to continuously reassess who does what, preventing overlap. Interview IZ1.

The purposes of publishing differ to food industry (see Penders and Nelis 2011), and it may have multiple audiences, as commercial researcher IZ1 explains: “[W]hen we do a clinical trial, then its purpose is also to allow marketing to work with it, immediately”. But beyond having different and multiple goals for academic writing, the organisation of scientific work in R&D departments of companies also creates different routes towards research publications. R&D researcher IB2 explains a small part of this path:

In dialogue with my boss, we decide whether to publish and decide how to distribute the work to do so. Big companies almost always have people who specifically do the writing. Sometimes, rarely, they may hire external writers. […] Not all projects lead to a publication and that doesn’t really matter for me. I do finish all of them with a final report. Interview IB2.

Research scientist IZ1 sketches a different organisation of work. IZ1 works at a significantly smaller company than IB2, which translates into a higher portion of IZ1’s company’s work being outsourced. IZ1 is involved in designing the study, but does not perform it. She does consult along the way:

We invited an external company to, ehm, perform the work for us. We are closely monitoring and supervising it. [...] In a recent project, we have outsourced a study, in which the outsourcing included data analysis and reporting. I am involved in talking to the statisticians on, ehm, how to best analyse the data. Interview IZ1.

When asked about how the publication trajectory of this study would look, IZ1 explained that in the writing process, which she expected not to perform herself, she would also be involved in a supervisory capacity, monitoring the company’s interest:

We also execute eh… external research, because we lack manpower to do it all internally. In that case, yeah, it mostly means that they also write it up. I would be there to supervise it, to see, eh, whether our company’s purpose for the publication are eh, met… for the most part. But it means that the research is done externally, and it is also written up externally. Interview IZ1.

She is not, as she admits, one of the intended authors of this study: “No, just like previously that is not the case”. She admits, neither are the external writers. Depending on where and in collaboration with whom the study was performed, other partners may be displayed in the authorship list in addition to her employer: “There is one colleague on there, representing [the company]”. Determining who got to represent the company was not particularly hard: “He was in charge”.

IZ1, a junior research scientist, is charged with controlling and managing research projects without actually performing them (or even without being where they are performed). She is involved in the analysis and the writing, in an advisory, contributory role, with the bulk of both tasks again, located elsewhere. However, she, just like many of the other researchers working on companies is performing other duties too. IZ1 spends a significant amount of her time answering questions originating elsewhere in the company, as well as by (prospective) customers, both individual and commercial. She negotiates the contours of new research projects with marketing departments and assists those same departments with compiling information leaflets and other promotional material.

Researcher IW5 similarly spends a lot of her time dealing with marketing issues, distributing scientific knowledge through the company and managing and controlling projects around new product development. She explains that new research ideas emerge together with new products: “Separate ingredients may have been tested before, but the product as a whole has not been tested. Or not for the target population we want to use it for”. She then instructs the in-house “scientific team” to do research. Often this would be literature research, hoping to avoid having to do research labour: “Ideally we would not do it ourselves, but if general researchers don’t do it, at some point, we’ll have to” (Interview IW5). Where IW5 identifies knowledge needs for the company, others cater to that need. When asked whether she read a lot of scientific literature, she answered:

No, we have a .. ehm. Because this is a big company, we have a team for that [...]. They have a certain specialisation and have been working in a field for a long time. They are the ones who read the most papers and assess whether we can use them or not. Of course, I read a paper on occasion [...]. I am spending most of my day organising practical stuff. Interview IW5.

Beyond the specialisation of literature research into a dedicated team, task division is identified by others as well, as relevantly different from academia:

Working for a company is comfortable, in a financial sense, but also because it provides some clarity in ehm, the way responsibilities are distributed. Jobs and tasks are clearly assigned. It is more clear and clearer from the start who does what, who is supposed to handle what and who is assessed based upon what. That also has its downside, I remember from academia, that when you, ehm…, do more than you should, something extra, it could also get you something extra in return… an authorship, a piece of a grant, contacts and so on. In a company this is more difficult. If you move beyond your mandate, you enter the responsibilities of others, especially when you are, like I am more recently, being hired externally. It is different. But I like it. Interview IB2.

That task division extends further, though, informed by different ways of organising work and different standards used in industrial settings. Researcher IU2 explain that in order to avoid industry research being discredited because of its origin, they do extra work and have to employ extra people to do it:

We always work according to the rules of good laboratory practice. There are external controls, say eh, audits, from our labs as well as other departments. That good laboratory practices requires additional administrative work, so there are special people to do it. Interview IU2.

Besides the need for additional people, as IU2 explains, different ways of organising work in for-profit research settings also translate into tasks that academic consider to ‘belong together’, being separated. With lots of people involved, deciding who becomes a co-author on a paper is however, remarkably easy, as research IU2 explains their company policy: “We do not want more than a certain number of people on a publication, regardless of work load […].” In collaborative work settings where IU2’s lab works together with an academic lab, this enables the apparent ownership of the study to start with the university, helping its credibility. Even though publication pressure is largely absent for individual researchers like IU2, this still results in frustration:

It is madness that you do most of the work, and that John or Pete from an external university, a person who did a lot less work, ends up on a more prominent position. That does not feel fair. Then you tell yourself, okay, I work at a company and we have that policy. Yes, then you’ll have to respect that. Interview IU2.

## Discussion

As one of their tasks, universities must teach junior academics how to be researchers. To this end, junior researchers are socialised into the ‘company’ culture and the tacit knowledge that accompanies the work – from performing experiments to research design and management to drafting and revising papers and more. The organisation of research work contributes to the situation that, for the most part, all academics engage in nearly all facets of research work. This includes the organisation of work into projects and the ways in which researchers are expected to communicate the results of those projects and the ways they are expected to shape their careers. Academic researchers perform many tasks and although these shift over the course of their careers, this still means that they are involved in research, handle data, write or contribute to publications and more. Consequently, they come close to the image invoked in authorship guidelines - to some degree.

In industry, the teaching role is largely absent. Of course, new employees are taught many things when they start work at a new firm, but the organisation of work differs in the sense that the array of tasks to be performed is a lot broader and the distribution of tasks across employees is organised differently. Since industrial researchers do not have to write to advance their careers, many of them do not engage in writing at all. They may produce informal memos or notes, provide requested data, maintain websites or more, but never draft a paper. Food companies do have a stake in academic publications (Penders and Nelis 2011) but this task is typically reserved for one or just a few people in the company, depending on the size. As a result, tasks are concentrated in individuals. As a collective, they can cover the entire spectrum. However, at the level of the individual, scientific work looks quite different. As a consequence, the majority of researchers working in the food industry do not qualify for authorship according to the ICMJE criteria in most papers being published.

However, that a lot of researchers working in the food industry do not qualify for authorship is not a major problem (for them). Their careers do not depend on getting authorship. The innovation and commercial agenda of the company requires authorship from some employees to be visible, but only from a select few. Yet, this does not mean that the misalignment between ICJME authorship criteria and the organisation of research in food industry is unproblematic.

Sismondo has demonstrated in his decade-long analysis of pharmaceutical publication practices that the pharmaceutical industry orchestrates both the conduct of the research and the ways it is used, for instance in the context of marketing (Sismondo 2007, 2009). In fact, Matheson (2011, 2016) has shown how this can take place within the confinements of ICMJE criteria: since approval of the final text is a requirement, medical writing firm employees can ensure that they do not (formally) qualify for authorship by voluntarily refusing to approve the final version. While the guidelines demand that all researchers involved should have the opportunity to participate in the approval of the manuscript, they are fully free to waive that opportunity.^4^ If the academic authors involved have contributed a little, for instance a few tips on research design and critical suggestions on a near-finished draft paper, they qualify as authors. Since the medical writers claim that they do not, the academics, often medical doctors, are left as the sole authors on the paper. The result is that the study is solely associated with an academic institution. The amount of backstage labour to produce this effect is easily compensated by the credibility benefit this brings to the study.

Similarly, some of the food industry publication practices may resemble the pharmaceutical model. A lot of work is done in collaboration with academic research partners especially because they confer credibility. The policy to minimise authorship that some companies have serves this purpose. A lot of labour is made invisible by the fact that research is organised differently. Different tasks are separated over multiple people and multiple experts, and writing, for instance, simply is not everyone’s job. This different organisation of work means that most industrial employees legitimately do not qualify for authorship. While formally this does not qualify as ghost authorship, there is a lot of ghost labour - labour made invisible by being organised differently than assumed in criteria like those articulated by ICMJE. These criteria do require acknowledgement of contributions that do not warrant authorship. However, this may also take place on a category level, acknowledging the support of a company or lab. Compared with the pharmaceutical industry, the food industry does not have as many researchers acting as key opinion leaders vouching for them and conferring credibility. Rather, by visibly participating in publication practices and by showing their company names and employees, they embed themselves in networks of nutrition researchers that have credibility - drawing from it, leaning on it.

The separation of different tasks across different departments and people was not designed to fool authorship criteria. The same goes for the involvement of more, other and different skills in the production of knowledge in the food industry, as well food industry decisions to outsource all sorts of labour to other companies or self-employed nutrition scientists. This way of organising knowledge production labour fits with what food industry seeks to achieve: structures to build legitimacy and credibility when faced by institutionalised distrust associated with profit. However, this does not mean that the fact that fewer employees qualify as authors does not conveniently allow policies such as the one quoted above: “We do not want more than a certain number of people on a publication, regardless of work load […].”

The differences in the governance of knowledge production between academia and industry translate into different ways in which they are represented on the by-lines of scientific literature. It downplays their involvement with published works, albeit not as extremely as in the pharmaceutical industry. It makes significant parts of the knowledge production work invisible or suggests that it was done elsewhere. This creates problems in terms of assigning responsibility and accountability. Finally, and more importantly, these differences show that authorship criteria are too narrowly articulated with a focus on an imagined practice that is itself not living up to them.

The pretence that industrial laboratories work like universities allows companies to hide behind universities and absorb their credibility. Unlike critical conceptualisations of undue corporate influence on science, such as *undone science* (Hess 2016), or *unseen science* (Richter et al. 2018), the credibility politics we describe concern articulating whose science this science is - not undone or unseen - but obscuring its provenance: *allonymous science*.^5^ Similarly, pretending that all academic scientists are renaissance women or men, or are five-legged sheep - to request unreasonable versatility, as the Dutch proverb goes - no longer holds up, if it ever did.^6^

The moral authority of authorship guidelines exists separate from their procedural gatekeeping role. This study joins others in questioning that moral authority (Kornhaber et al. 2015; Macfarlane 2017; Shaw 2011), given that the organisation of research labour limits the potential of compliance and reasserts existing power distribution in research. As long as the misalignment of practice and guidelines endures, we will see them broken, misused or abused. Allonymous science - the way this misalignment manifests itself on the by-lines of scientific papers - is difficult to prevent, since it is a manifestation of synergetic interests in the context of public-private collaboration. Cultures of reward in both academia and industry actively contribute to its maintenance, and while evolving these cultures may offer a way out in the long run, they are unlikely to make allonymous science disappear quickly.

## Conclusion

The expectations of the imagined researcher and the imagined organisation of research, embedded in the ICMJE authorship rules, are based on the social organisation of science in academia. Yet, even in academia, living up to the ‘renaissance’ norms is very difficult. While many and perhaps most researchers make some attempt to accomplish this near impossible task, power asymmetries and systemic pressures render actual research practices obsolete. Despite these difficulties, the organisation of research, most notably in the form of the distribution of tasks and responsibilities across people, at least would allow most researchers in academia to pursue and approach compliance to ICMJE criteria for authorship.

The social and epistemic organisation of science in industry is quite different, especially when it comes to the distribution of tasks and responsibilities across people. As a consequence, the actual research work and practice, as well as the organisation of research work, do not align with assumptions in authorship guidelines. Industry does not argue that this is unjust, unfair or leads to discontent or defiance. Instead, the food industry complies with the letter of the rules, thereby allowing fewer of its researchers to become authors. This compliance is used instrumentally, enabling allonymous science: the suggestion that the presented science is, largely or partially, someone else’s science. This allows private actors to absorb credibility from public knowledge institutions and centralise credibility in a few of their people.

A shift from authorship to contributorship has been proposed to tackle the “writing mandatory part” of authorship requirements (e.g. Holcombe (2019), yet the subdivision of activities and tasks as listed in the credit system is still based rooted in the same expectations of the imaged researcher, research labour and research governance. While this would shift many of the bases, it would be unlikely to alleviate all issues across sectoral borders. Similarly, asking all authors to legitimate or defend their own authorship would do little to make visible those who voluntarily opt out of authorship (Malički et al. 2012). If we choose to continue to treat publications in the same way, regardless of source, and bestow value and credibility accordingly, we need to acknowledge the different and sometimes competing organisational structures that bring such products into existence. Revealing the dynamics behind allonymous science helps us interpret (or read between the lines) of academic bylines. Any solution or alleviating measure will need to situate itself contextually within the realignment of the relevant organizational structures and credit distributing practices our paper identifies and explores, and would have to seek input from those occupying the public-private borderlands in nutrition science and elsewhere. This would imply that we need to move away from university-centric views on the organisation of knowledge-making This would allow credit to be assigned in multiple ways and would also acknowledge how such credit is assigned rather than employ unrealistic notions of the “renaissance” researcher who is difficult or impossible to find, yet is imagined to occupy desks and laboratories everywhere.

## Data Availability

In accordance with the ethical permission received, and the personal nature of all qualitative interview transcripts, no data can be shared. Data is available for auditing purposes under the Maastricht University, Care and Public Health Research Institute (CAPHRI) Research Quality Assessment frame. All qualitative data was collected, handled and stored in agreement with the Maastricht University Data Management Code-of-Conduct.

## Footnotes

1 Elsewhere, in an earlier publication on the 2016 interview series discussing the social dynamics of credit distribution and valuation, respondent selection is discussed in greater detail (Penders 2017a).

2 The gatekeeping infrastructure can take different forms. Often, the corresponding author is required to claim in testify the he or she, as well as all other authors, have lived up the guidelines. Alternatively, each author can be required to testify that she or he upheld the requirements individually. These testimonials are a prerequisite for access to journal publication.

3 Note that this criterion assumes that data is involved in some way, an assumption that neglects the important role played by analytical/theoretical work.

4 We would argue that refusing to endorse a final version for this technical reason when one presumably does actually endorse it may be compatible with the letter of the guidelines, but not their spirit.

5 Allonymous science here is an addition to the repertoire of strategies to form the face of science that food industry can employ - deliberately or less so.

6 Shapin (1989) for instance, shows how the organisation of laboratories allowed appropriation of technician’s labour throughout history.
